# Haplotype Analysis Reveals a Possible Founder Effect of *RET* Mutation R114H for Hirschsprung's Disease in the Chinese Population

**DOI:** 10.1371/journal.pone.0010918

**Published:** 2010-06-02

**Authors:** Belinda K. Cornes, Clara S. Tang, Thomas Y. Y. Leon, Kenneth J. W. S. Hui, Man-Ting So, Xiaoping Miao, Stacey S. Cherny, Pak C. Sham, Paul K. H. Tam, Maria-Merce Garcia-Barcelo

**Affiliations:** 1 Paediatric Surgery Division, Department of Surgery, Li Ka Shing Faculty of Medicine, University of Hong Kong, Pokfulam, Hong Kong, Special Administrative Region, People's Republic of China; 2 Department of Psychiatry, Li Ka Shing Faculty of Medicine, University of Hong Kong, Pokfulam, Hong Kong, Special Administrative Region, People's Republic of China; Ohio State University Medical Center, United States of America

## Abstract

**Background:**

Hirschsprung's disease (HSCR) is a congenital disorder associated with the lack of intramural ganglion cells in the myenteric and sub-mucosal plexuses along varying segments of the gastrointestinal tract. The *RET* gene is the major gene implicated in this gastrointestinal disease. A highly recurrent mutation in *RET* (*RET^R114H^*) has recently been identified in ∼6–7% of the Chinese HSCR patients which, to date, has not been found in Caucasian patients or controls nor in Chinese controls. Due to the high frequency of *RET^R114H^* in this population, we sought to investigate whether this mutation may be a founder HSCR mutation in the Chinese population.

**Methodology and Principal Findings:**

To test whether all *RET^R114^* were originated from a single mutational event, we predicted the approximate age of *RET^R114H^* by applying a Bayesian method to *RET* SNPs genotyped in 430 Chinese HSCR patients (of whom 25 individuals had the mutation) to be between 4–23 generations old depending on growth rate. We reasoned that if *RET^R114H^* was a founder mutation then those with the mutation would share a haplotype on which the mutation resides. Including SNPs spanning 509.31 kb across *RET* from a recently obtained 500 K genome-wide dataset for a subset of 181 patients (14 *RET^R114H^* patients), we applied haplotype estimation methods to determine whether there were any segments shared between patients with *RET^R114H^* that are not present in those without the mutation or controls. Analysis yielded a 250.2 kb (51 SNP) shared segment over the *RET* gene (and downstream) in only those patients with the mutation with no similar segments found among other patients.

**Conclusions:**

This suggests that *RET^R114H^* is a founder mutation for HSCR in the Chinese population.

## Introduction

Hischsprung's disease (HSCR) is a congenital disorder associated with the lack of intramural ganglion cells in the myenteric and sub-mucosal plexuses along varying segments [short-segment aganglionosis (S-HSCR), long-segment aganglionosis (L-HSCR) and total colonic aganglionosis (TCA)] of the gastrointestinal tract. There is significant racial variation in the incidence of the disease and it is most often found among Asians (28 per 100,000 live births) [1], [2]. The male to female ratio (M∶F) is ∼4∶1 among S-HSCR patients and ∼1∶1 among L-HSCR patients. The recurrence risks to siblings vary from 1.5 to 33% depending on gender and length of the aganglionic segment in the proband and the gender of the sibling. In general, HSCR presents mostly sporadically, although it can be familial with a complex pattern of inheritance, including low, sex-dependent penetrance and phenotypic variability. HSCR has also a complex genetic aetiology; many studies have indicated receptor tyrosine kinase (*RET*) as the major susceptibility gene for HSCR [3], [4]. Mutations in the coding regions of *RET* account for over 50% of the familial cases, and between 7%–35% of the sporadic cases. *RET* mutations are not fully penetrant indicating that the disease may result from the combined effect of *RET* and other interacting disease susceptibility alleles.

Some disorders are characterised by a mutation(s) that can be traced back to a founder and whose existence can be inferred from the unique chromosomal background on which the mutation occurred. Some founder mutations may be a major cause of the disorder, because they lead to a specific phenotype–the disease occurs only if that mutation is present. Alternatively, there is no specific correlation with phenotype but the reproductive history within a given population led to a relatively high frequency for the mutation and, thus, to its becoming a major cause of a disease.

Genetic mapping, family based association studies, comparative sequence analysis and functional studies cumulatively provided evidence for a common polymorphism (rs2435357: C→T) in intron 1 of *RET* that is the strongest contributor to HSCR risk [5], [6]. This SNP is common among different populations including Asians. Additionally it has been demonstrated that HSCR European and Chinese patients share the same background haplotype containing this high risk SNP although the frequency of the disease haplotype is higher in Chinese than in Europeans [7], [8].

Thus far, over 200 non recurrent *RET* coding sequence (CDS) mutations have been identified in HSCR patients. In a 2004 study [9], a highly recurrent (6–7% of the patients) heterozygous missense mutation was identified in the extracellular domain (exon 3) of the *RET* protein (*RET^R114H^* mutation; c.341G>A according to NP_066124.1 and NM_020975.4 respectively). The frequency of *RET^R114H^* has remained stable after having sequenced 262 additional Chinese HSCR patients [10]. This mutation was not found in Chinese controls (*N* = 224) nor has it been found, to date, in Caucasian HSCR cases or controls. The high recurrence of *RET^R114H^* in Chinese HSCR patients lead us to investigate whether its frequency is due to a possible mutational hot spot (i.e. arisen independently) or, perhaps, a possible founder mutation for a sub-population of HSCR patients whose significance is not yet known.

Haplotype studies could help to determine if a specific mutation appears to occur on a common genetic background suggesting it has been inherited from a common ancestor, or if the shared mutation appears to have arisen independently multiple times. In the current study, we use haplotype methods in a case-control Chinese HSCR sample to determine the relative importance of the *RET^R114H^* mutation in the evolution of HSCR in Asia.

## Materials and Methods

### Subjects and Measures

Patients and their families were referred by family members or their physicians. Their participation as well as the participation of healthy controls was conditional upon informed written consent under the ethical guidance of the Institutional Review Board of The University of Hong Kong together with the Hospital Authority (IRB: UW06-349 T/1374). A total of 430 Chinese HSCR patients and 632 Chinese controls were included in the study. Diagnosis of HSCR was based on histologic examination of either biopsy or surgical resection material for absence of enteric plexuses. Blood samples were drawn from all patients after obtaining informed written consent but where participants were newborns or under the age of seven, parental consent was obtained instead.

#### RET SNP genotyping and sequencing

All participants (430 patients and 224 controls) were genotyped for 21 *RET* SNPs across a ∼60 kb region of *RET* as well as sequenced for the 21 *RET* exons. See supplementary table, Table S1, for a further description of these SNPs. DNA for genotyping was extracted from peripheral blood using a Qiagen kit. Genotyping was carried out by primer extension (MassEXTEND), and MALDI-TOF (Matrix Assisted Laser Desorption Ionization-Time Of Flight) mass spectrometry as the read-out system on a Sequenom platform (Sequenom MassARRAY system, Sequenom, San Diego CA), according to the manufacturer's instructions and as previously described in Garcia-Barcelo et al. [11]. Sequencing of *RET* exons was performed as previously described. Among the 430 HSCR patients, the *RET^R114H^* mutation [9] was identified in 25 (∼6%) patients.

All 21 SNPs tested had a call rate of over 95% and were in Hardy-Weinberg equilibrium after correcting for multiple testing. All SNPs, except rs302678, were polymorphic.

#### 500 K Genome-wide Scan

Forty six additional SNP genotypes encompassing the *RET* gene ±250 kb were obtained through a genome-wide association study (GWAS) conducted on 181 HSCR patients selected from the original sample of 430 HSCR patients. For this GWAS, controls consisted of 306 individuals (106 males, 200 females) of southern Chinese origin participating in an ongoing GWA study aimed at the discovery of genetic factors for the quantitative trait of disk degeneration (DD) (see Garcia-Barcelo et al. [12]) for further information) as well as a derived synthetic control set formed by combining the non-transmitted alleles from some of the parents of HSCR cases, or “pseudo-controls” (*N* = 57), and *Affymetrix* generated genotypes for the Chinese Han population (*N* = 45) retrieved from the International HapMap Project. This provided a total control sample of 408 individuals for this GWA study.

### Statistical Data Analyses

#### Haplotype Analysis

Haplotypes were estimated using the statistical software package PHASE version 2.1 [http://www.stat.washington.edu/stephens/; [13], [14], [15]], a program based on a Bayesian statistical method using coalescent-based models that infers phases at loci from unphased genotype data for a sample of unrelated individuals [13], though extensions to related individuals are possible [16]. The algorithm uses a flexible model for the decay of linkage disequilibrium with distance and explicitly incorporates an assumption about recombination rate variation. PHASE uses Gibbs sampling, a Markov-Chain Monte Carlo algorithm [17] for the estimation of the posterior distribution. Hence, the individual haplotype can be estimated from the posterior distribution by choosing the most likely haplotype reconstruction for each individual.

Using the extension for unrelated individuals, we used the default settings for recombination rate variation [18] to infer the haplotypes from the genotype data of the 21 SNPs and the *RET^R114H^* mutation (used as a SNP in the analysis) for 430 patients (25 with *RET^R114H^* mutation) and again for the additional 46 SNPs added to the sample (*N* = 68 SNPs) for a subsample of 181 cases (14 of whom have the *RET^R114H^* mutation). Estimates of the sample haplotype frequencies together with their standard deviation, a list of the most likely pairs of haplotypes for each individual together with their probability, and the estimates of recombination parameters in the region, were calculated using the same software under the assumption of a stepwise mutation mechanism for multiallelic loci.

#### Age of Mutation Analysis

DMLE+ version 2.2 developed by Reeve and Rannala (2002) [19] (http://dmle.org/) was used to estimate the age of the *RET^R114H^* mutation. This program was designed for high-resolution mapping of a disease mutation and estimation of its age. The method is based on the observed linkage disequilibrium between a disease mutation and linked markers in DNA samples of unrelated normal individuals and affected patients. The program uses the Markov Chain Monte Carlo algorithm to allow Bayesian estimation of the mutation age based on the following parameters: the observed genotypes (or haplotypes) in samples of unrelated normal or affected chromosomes, map distances between markers and the position of the mutation relative to the markers and the estimated population growth rate. Simulation analyses have shown that good results are obtainable even with a fair degree of genetic heterogeneity and phenocopy, and incomplete penetrance [20].

In addition to the genotypic data, marker (or SNP) location, population growth rate, and an estimate for the proportion of disease bearing chromosomes being analysed are used by the software. We used recommended burn-in and sampling intervals and a variety of modeling assumptions and parameter ranges. In this study the parameters used were the haplotypes generated from 21 SNPs in unrelated normal and affected individuals (as this information was available for the most number of cases and controls), the chromosome map distances between markers and disease mutations, and 0.02834% mean carrier frequency of *RET^R114H^* mutation (estimated from number of disease chromosomes in sample). For the population growth rate, we took data from the Hong Kong Census and Statistics Department (http://www.censtatd.gov.hk/home/index.jsp), which records population figures back to 1961. The department records show that, on average, the population growth rate has decreased with time; so to account for this, we used an average growth rate across the whole time period (1.7%) as well as using two extreme growth rates on record–the highest growth rate recorded was 5.4% in 1962 and the lowest growth rate was 0.3% in 2001. To compare results, we also performed the estimation using the 2007 growth rate (0.606%).

One disadvantage with the program is that it does not allow information about the mutation genotype (in the disease chromosomes) and the location of the mutation to be used simultaneously. Therefore, the program was run once with mutation location specified and with no genotype information included and once with genotype information and mutation location freed. Both returned similar results.

## Results and Discussion

### Preliminary Haplotype Analysis

**Figure 1:**
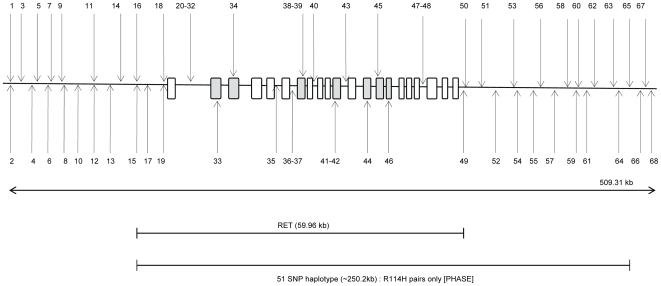
Schematic diagram (not to scale) showing the location of SNPs used in the analysis across the *RET* gene. The exons and promoter are represented by rectangles (a darker grey is used for those in which a SNP is located). See Table S1 for the name of each SNP represented in this figure.

To determine whether all occurrences of *RET^R114H^* mutation descended from a single ancestral mutation event or arisen independently, we first constructed haplotypes with 21 *RET* region SNP genotypes from 224 controls and 430 HSCR patients of whom 25 were *RET^R114H^* mutation carriers (see Figure 1).

Haplotype reconstruction, carried out using the statistical software PHASE v2.1.1, suggested that 61 different haplotypes exist in the current sample. The software also revealed that every patient harbouring *RET^R114H^* was predicted with a probability over 70% to have a common mutation haplotype (T-G-A-C-A-A-A-**T**-A-G-A-**M**-C-G-G-G-C-G-G-C-C-C) which comprises the HSCR-associated allele T of the intron 1 SNP rs2435357 [5] [**M** represents the allele change from the wild type c.341G>A for *RET^R114H^*; **T** represents the high risk SNP (rs2435357) in intron 1 of *RET*] (see Table 1). Furthermore, 293 out of the rest of the 406 non-carrier cases (72.17%) were predicted to carry the same haplotype without the mutation (i.e. T-G-A-C-A-A-A-**T**-A-G-A-**G**-C-G-G-G-C-G-G-C-C-C). Overall, this haplotype accounted for the largest proportion in the sample. This result was not surprising given that early results predicted that HSCR patients are predominantly represented by the same haplotype [8]. However, we reasoned that if this mutation had a single ancestral origin, carriers would have more chromosomal segments in common than non-carrier patients.

**Table 1:** Frequencies of the different haplotypes in HSCR cases with and without the *RET^R114H^* mutation.

														*RET* starts																
rs2795500	rs2744088	rs2744085	rs12768318	rs3121323	rs2488291	rs11239832	rs788273	rs788261	rs7908085	rs2185792	rs10900290	rs947699	rs2082106	rs3026720*	rs741763*	rs2505995	rs10900296*	rs10900297*	rs2506011	rs1864410*	rs2435364*	rs2435362*	rs2435357*	rs2435356	rs2506021	rs2435342	rs752975*	rs2505538	rs2505535*	rs2505533
**21 SNPs plus R114H sequencing data haplotype estimates:**																														
														T	G		A	C		A	A	A	T				A		G	
**67 SNPs plus R114H genome-wide and sequencing data haplotype estimates:**																														
														T	G	T	A	C	A	A	A	A	T	A	G	T	A	T	G	A
														T	G	T	A	C	A	A	A	A	T	A	G	T	A	T	G	A
														T	G	T	A	C	A	A	A	A	T	A	G	T	A	T	G	A
														T	G	T	A	C	A	A	A	A	T	A	G	T	A	T	G	A
A	G	C	A	G	A	G	T	G	A	G	C	A	C	T	G	T	A	C	A	A	A	A	T	A	G	T	A	T	G	A
A	G	C	A	G	A	G	T	G	A	G	C	A	C	T	G	T	A	C	A	A	A	A	T	A	G	T	A	T	G	A
A	G	C	A	G	A	G	T	G	A	G	C	A	C	T	G	T	A	C	A	A	A	A	T	A	G	T	A	T	G	A
A	G	C	A	G	A	G	T	G	A	G	C	A	G	T	G	T	A	C	A	A	A	A	T	A	G	T	A	T	G	A
A	G	C	A	G	A	G	T	G	A	G	C	A	G	T	G	T	A	C	A	A	A	A	T	A	G	T	A	T	G	A
A	G	C	A	G	A	G	T	G	A	G	C	A	G	T	G	T	A	C	A	A	A	A	T	A	G	T	A	T	G	A
A	G	C	A	G	A	G	T	G	A	G	C	A	G	T	G	T	A	C	A	A	A	A	T	A	G	T	A	T	G	A
A	G	C	A	G	A	G	T	G	A	G	C	A	G	T	G	T	A	C	A	A	A	A	T	A	G	T	A	T	G	A

																		*RET* ends												
rs3123655	rs1800858*	R114H*	rs2565205*	rs1864403	rs2251674	rs1800860*	rs9282834*	rs3026750*	rs55862116*	rs1799939*	rs2742234	rs1800861*	rs1800862*	rs1800863*	rs2742236	rs2742237*	rs2565200	rs17028	rs3004214	rs2505506	rs7921281	rs7893332	rs7092548	rs2435377	rs12220534	rs1879310	rs7093409	rs7090455	rs2505526	rs2503872rs2493650 rs11238510 rs2503875 rs2493654 rs2460535 rs2243492% shared in Cases [without R114H]% shared in Cases [with R114H]
																														
	A	M	C			G	G	G	C	G		G	C	C		C														72.17% 100%
																														
G	A	M	C	T	G	G	G	G	C	G	G	G	C	C	G	C	A	C	C	C	T	T	C	G	G	G	G	A	G	GG G T 44.31% 100%
G	A	M	C	T	G	G	G	G	C	G	G	G	C	C	G	C	A	C	C	C	T	T	C	G	G	G	G	A	G	GG G T C 35.33% 71.43%
G	A	M	C	T	G	G	G	G	C	G	G	G	C	C	G	C	A	C	C	C	T	T	C	G	G	G	G	A	G	GG G T C G T 10.78% 50.00%
G	A	M	C	T	G	G	G	G	C	G	G	G	C	C	G	C	A	C	C	C	T	T	C	G	G	G	G	A	G	GG G T C A C 24.55% 21.43%
G	A	M	C	T	G	G	G	G	C	G	G	G	C	C	G	C	A	C	C	C	T	T	C	G	G	G	G	A	G	GG G T C 5.39% 35.71%
G	A	M	C	T	G	G	G	G	C	G	G	G	C	C	G	C	A	C	C	C	T	T	C	G	G	G	G	A	G	GG G T C G T 1.80% 28.57%
G	A	M	C	T	G	G	G	G	C	G	G	G	C	C	G	C	A	C	C	C	T	T	C	G	G	G	G	A	G	GG G T C A C 3.59% 7.14%
G	A	M	C	T	G	G	G	G	C	G	G	G	C	C	G	C	A	C	C	C	T	T	C	G	G	G	G	A	G	GG G T 0% 64.29%
G	A	M	C	T	G	G	G	G	C	G	G	G	C	C	G	C	A	C	C	C	T	T	C	G	G	G	G	A	G	GG G T C 0% 35.72%
G	A	M	C	T	G	G	G	G	C	G	G	G	C	C	G	C	A	C	C	C	T	T	C	G	G	G	G	A	G	GG G T C G T 0% 21.43%
G	A	M	C	T	G	G	G	G	C	G	G	G	C	C	G	C	A	C	C	C	T	T	C	G	G	G	G	A	G	GG G T C A C 0% 14.29%
G	A	M	C	T	G	G	G	G	C	G	G	G	C	C	G	C	A	C	C	C	T	T	C	G	G	G	G	A	G	GG G T A A C 0% 28.57%

*genotyped as part of original sample.

M = mutant allele (A).

### Age of Mutation

**Figure 2:**
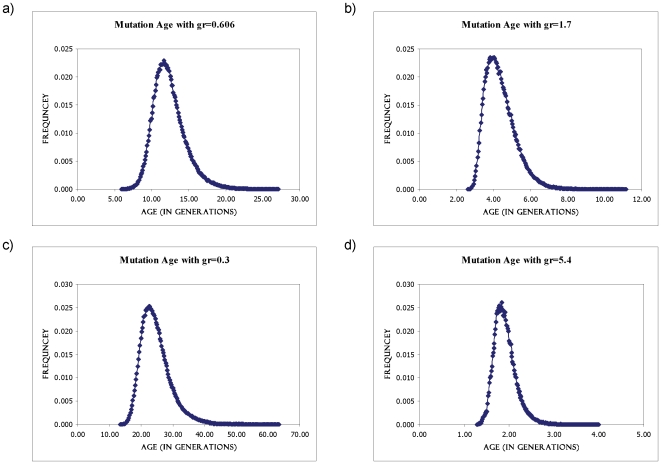
Age estimates for the *RET^R114H^* mutation. The posterior probability distribution plots of the mutation age (in generations), as estimated by the software DMLE+2.2, are shown with population growth rates of 0.606 (a), 1.7 (b), 0.3 (c) and 5.4 (d).

Using the above set of individuals and data, we estimated the “age” of *RET^R114H^* assuming that it has originated from a common ancestor. The decay of linkage disequilibrium (LD) due to recombination can be used to date the age of the mutation or that of its introduction into the analysed population on a particular haplotype. We used the DMLE+2.2 software to estimate the age of the mutation using haplotype data from affected patients and unaffected controls. Figure 2 shows the posterior probability densities of the mutation age for *RET^R114H^* for the four different population growth rates.

For the first analysis, which used the most up-to-date growth rate estimated, the mutation density depicted a peak at ∼11–12 generations for *RET^R114H^*. Assuming 20–30 years for a generation, the age of the *RET^R114H^* mutation was estimated to be between 220–340 years old. The second analysis, which used an average growth rate, gave an age estimate of 80–120 years (∼4 generations). The third analysis used the slowest growth rate on record and gave an age estimate of 440–690 years (∼22–23 generations). The last analysis, which used the fastest growth rate on record, gave an age estimate of ∼36–54 years (∼1.8 generations).

The analysis of the extent of decay of LD between the mutation and the adjacent SNPs suggests that the founder effect may easily explain the high frequency of the mutation in the Chinese. However, although *RET^R114H^* is on the haplotype background shared by Caucasians and Chinese [5], [8], had the mutation occurred before the European-Asian split, its frequency in our population would be much higher than that currently observed. Thus either the mutation is in fact a founder mutation for a sub-population of Chinese HSCR or more than one carrier has introduced the mutation into the Chinese population given its relatively young age.

### Extended Haplotype Sharing

We reasoned that if *RET^R114H^* had a single ancestor origin, carriers would have more chromosomal segments in common than non-carrier patients. In order to support our hypothesis, we made use of a subset of samples that had previously been genotyped for a GWAS (181 patients of whom 14 carry the *RET^R114H^* heterozygous mutation) [12]. Before performing haplotype analysis on this subsample, we set about to determine whether there were any groups of individuals (in particular those with the *RET^R114H^* mutation) that are more related to each other than to other cases and/or controls. Multidimensional scaling (MDS) in PLINK version 1.03 [21] was used, which is a set of related statistical techniques often used to visualize for patterns of subgroupings in data. There was no clustering of *RET^R114H^* individuals indicating that no specific similarities were shared more/less between these individuals at the whole genome level (results not shown).

To be able to include as much information as possible to increase power for haplotype estimation, we isolated and merged 46 SNPs located on or within the region of 250 kb of *RET* from the 500 K genome-wide dataset; therefore PHASE was conducted on a total sample of 68 SNPs (including the *RET^R114H^* mutation) across a 509.31 kb region across *RET* in a total of 181 patients (14 *RET^R114H^* mutation) that were included in both the original dataset and 500 K genome-wide dataset. Descriptive statistics of these *RET* SNPs are shown in the supplementary table, Table S1. Figure 1 shows a Schematic diagram of the combined *RET* SNPs while Table 1 shows haplotype frequencies estimated by the PHASE program.

PHASE analysis performed on the 68 SNP sample yielded a 250.2 kb (51 SNPs) shared segment over the *RET* gene (and downstream) in only *RET^R114H^* patients with no similar segments found among non-mutant carriers (see Table 1). This demonstrates that HSCR patients with the *RET^R114H^* mutation have a common ancestor. The fact that the haplotype of the *RET^R114H^* patients is longer than that of the other non-mutant patients is in line with the “young” age of the mutation and lower recombination across this haplotype. However, the length of the segment shared between mutant carriers suggest that the mutation may be older than what has been predicted. In previous studies, it has been suggested that there are possible recombination events between intron 5 and intron 8 of the *RET* gene causing a risk haplotype frequency difference between Caucasians and Chinese originating in two “risk haplotypes”. It has also been suggested that the reason for these differences between populations is that the true *RET* disease susceptibility allele remains to be identified. Therefore, the *RET^R114^* ^H^ mutation may be a recent (say, a few centuries) random event that may or may not be in LD with the true *RET* causal variant given its relatively young age and its location on the ‘risky’ haplotype. This suggestion is not unlikely given the high LD along *RET* [8], [22] and the rarity of the mutation.

In conclusion, this study shows that the *RET^R114H^* mutation is likely a founder mutation in a subset of Chinese HSCR patients. We could not find any correlation between the mutation and the severity of the phenotype or gender (two major risk factors) which is somehow expected given the genetic complexity of the condition. Yet, the fact that *RET^R114H^* has never been found in controls and given its high frequency in the Chinese population, it is tempting to speculate that indeed *RET^R114H^* increases the risk to disease. Therefore, functional studies to uncover the effect of *RET^R114H^* on the gene or protein function are warranted.

## Supporting Information

Table S1 Descriptive Statistics of SNPs included in the current study.
(0.18 MB DOC)
