# Supplementary material for: Haplotype Analysis Reveals a Possible Founder Effect of RET Mutation R114H for Hirschsprung's Disease in the Chinese Population
Source: PLoS One. 2010 Jun 2;5(6):e10918. doi: 10.1371/journal.pone.0010918 (PMC2880000; doi:10.1371/journal.pone.0010918)
Supplement: Table S1 — Descriptive Statistics of SNPs included in the current study. (0.18 MB DOC) [file pone.0010918.s001.doc]

| **SNP #** | **SNP name** | **Position** | **Gene** | **Gene Region** | **Alleles** | **Minor Allele Frequency** | | **Allele Frequency dbSNP (HapMap HCB)** | **Dataset** |
| --- | --- | --- | --- | --- | --- | --- | --- | --- | --- |
|  |  |  |  |  |  | **HSCR** | **controls** |  |  |
| 1 | rs2795500 | 42660051 | - | - | C/T | C: 0.126 | C: 0.134 | C: 0.122 T: 0.878 | 500K genome-wide |
| 2 | rs2744088 | 42660136 | - | - | A/G | A: 0.131 | A: 0.139 | A: 0.122 G: 0.878 | 500K genome-wide |
| 3 | rs2744085 | 42661982 | - | - | C/T | T: 0.133 | T: 0.137 | C: 0.878 T: 0.122 | 500K genome-wide |
| 4 | rs12768318 | 42676583 | - | - | A/T | T: 0.494 | A: 0.483 | A: 0.522 T: 0.478 | 500K genome-wide |
| 5 | rs3121323 | 42697172 | - | - | A/G | A: 0.128 | A: 0.136 | A: 0.122 G: 0.878 | 500K genome-wide |
| 6 | rs2488291 | 42738999 | - | - | C/T | C: 0.310 | C: 0.230 | C: 0.278 T: 0.722 | 500K genome-wide |
| 7 | rs11239832 | 42739373 | - | - | A/C | A: 0.137 | A: 0.203 | A: 0.200 C: 0.800 | 500K genome-wide |
| 8 | rs788273 | 42750320 | - | - | A/T | A: 0.067 | A: 0.102 | A: 0.078 T: 0.922 | 500K genome-wide |
| 9 | rs788261 | 42757976 | - | - | C/T | T: 0.381 | T: 0.235 | C: 0.778 T: 0.222 | 500K genome-wide |
| 10 | rs7908085 | 42780789 | - | - | A/T | T: 0.034 | T: 0.204 | A: 0.167 T:0.833 | 500K genome-wide |
| 11 | rs2185792 | 42780880 | - | - | A/G | A: 0.094 | A: 0.277 | A: 0.333 G: 0.667 | 500K genome-wide |
| 12 | rs10900290 | 42791581 | - | - | C/T | T: 0.039 | T: 0.274 | - | 500K genome-wide |
| 13 | rs947699 | 42813903 | - | - | A/G | A: 0.003 | A: 0.416 | A: 0.367 G: 0.633 | 500K genome-wide |
| 14 | rs2082106 | 42852037 | - | - | C/G | G: 0.198 | G: 0.317 | C: 0.611 G: 0.389 | 500K genome-wide |
| 15 | rs3026720 | 42883860 | *RET* | 5'UTR | C/T | C: 0.041 | C: 0.159 | C: 0.100 T: 0.900 | Original sample |
| 16 | rs741763 | 42888352 | *RET* | 5'UTR | C/G | C: 0.086 | C: 0.371 | C: 0.344 G: 0.656 | Original sample |
| 17 | rs2505995 | 42889659 | *RET* | 5'UTR | A/G | G: 0.406 | G: 0.349 | A: 0.656 G: 0.344 | 500K genome-wide |
| 18 | rs10900296 | 42892513 | *RET* | 5'UTR | A/G | G: 0.139 | A: 0.429 | - | Original sample |
| 19 | rs10900297 | 42892517 | *RET* | 5'UTR | A/C | A: 0.076 | A: 0.377 | - | Original sample |
| 20 | rs2506011 | 42894942 | *RET* | intron 1 | C/T | C: 0.084 | C: 0.351 | C: 0.344 T: 0.656 | 500K genome-wide |
| 21 | rs1864410 | 42895631 | *RET* | intron 1 | A/C | C: 0.158 | A: 0.426 | - | Original sample |
| 22 | rs2435364 | 42896255 | *RET* | intron 1 | C/T | C: 0.089 | C: 0.374 | - | Original sample |
| 23 | rs2435362 | 42898785 | *RET* | intron 1 | G/T | G: 0.147 | T: 0.440 | - | Original sample |
| 24 | rs2435357 | 42902062 | *RET* | intron 1 | A/G | G: 0.139 | A: 0.411 | - | Original sample |
| 25 | rs2435356 | 42903156 | *RET* | intron 1 | C/T | C: 0.149 | T: 0.471 | C: 0.489 T: 0.511 | 500K genome-wide |
| 26 | rs2506021 | 42904154 | *RET* | intron 1 | C/T | T: 0.087 | T: 0.347 | C: 0.682 T: 0.318 | 500K genome-wide |
| 27 | rs2435342 | 42904262 | *RET* | intron 1 | C/T | C: 0.139 | C: 0.429 | C: 0.364 T: 0.636 | 500K genome-wide |
| 28 | rs752975 | 42908319 | *RET* | intron 1 | A/G | G: 0.127 | G: 0.446 | A: 0.568 G: 0.432 | Original sample |
| 29 | rs2505538 | 42911409 | *RET* | intron 1 | C/T | C: 0.144 | C: 0.434 | C: 0.378 T: 0.622 | 500K genome-wide |
| 30 | rs2505535 | 42913049 | *RET* | intron 1 | C/T | T: 0.184 | C: 0.417 | C: 0.456 T: 0.544 | Original sample |
| 31 | rs2505533 | 42914459 | *RET* | intron 1 | A/G | C: 0.189 | A: 0.465 | A: 0.568 G: 0.432 | 500K genome-wide |
| 32 | rs3123655 | 42915200 | *RET* | intron 1 | C/G | C: 0.131 | C: 0.349 | C: 0.322 G: 0.678 | 500K genome-wide |
| 33 | rs1800858 | 42915974 | *RET* | exon 2 | A/G | G: 0.179 | A: 0.411 | A: 0.556 G: 0.444 | Original sample |
| 34 | R114H | 42917800 | *RET* | exon 3 |  |  |  | N/A | Original sample |
| 35 | rs2565205 | 42923018 | *RET* | intron 5 | C/G | G: 0.041 | G: 0.147 | - | Original sample |
| 36 | rs1864403 | 42924956 | *RET* | intron 6 | C/T | C: 0.202 | T: 0.463 | C: 0.444 T: 0.556 | 500K genome-wide |
| 37 | rs2251674 | 42925398 | *RET* | intron 6 | A/G | A: 0.030 | G: 0.452 | A: 0.444 G: 0.556 | 500K genome-wide |
| 38 | rs1800860 | 42926693 | *RET* | exon 7 | A/G | A: 0.098 | A: 0.182 | A: 0.148 G: 0.852 | Original sample |
| 39 | rs9282834 | 42926862 | *RET* | exon 7 | A/G | A: 0.037 | A: 0.020 | A: 0.023 G: 0.977 | Original sample |
| 40 | rs3026750 | 42927762 | *RET* | intron 8 | A/G | A: 0.189 | G: 0.473 | - | Original sample |
| 41 | rs55862116 | 42930091 | *RET* | exon 11 | C/T | T: 0.004 | T: 0.005 | - | Original sample |
| 42 | rs1799939 | 42930125 | *RET* | exon 11 | A/G | A: 0.058 | A: 0.263 | A: 0.144 G: 0.856 | Original sample |
| 43 | rs2742234 | 42932615 | *RET* | intron 12 | C/T | T: 0.006 | C: 0.481 | C: 0.556 T: 0.444 | 500K genome-wide |
| 44 | rs1800861 | 42933849 | *RET* | exon 13 | A/C | A: 0.069 | A: 0.491 | A: 0.521 C: 0.479 | Original sample |
| 45 | rs1800862 | 42935100 | *RET* | exon 14 | C/T | T: 0.002 | C: 1.000 | - | Original sample |
| 46 | rs1800863 | 42935639 | *RET* | exon 15 | C/G | G: 0.035 | G: 0.101 | C: 0.856 G: 0.144 | Original sample |
| 47 | rs2742236 | 42940557 | *RET* | intron 18 | A/G | A: 0.037 | A: 0.303 | A: 0.244 G: 0.756 | 500K genome-wide |
| 48 | rs2742237 | 42941329 | *RET* | intron 18 | C/G | G: 0.186 | C: 0.449 | C: 0.531 G: 0.469 | Original sample |
| 49 | rs2565200 | 42942939 | *RET* | 3'UTR | A/G | G: 0.188 | A: 0.484 | A: 0.556 G: 0.444 | 500K genome-wide |
| 50 | rs17028 | 42943818 | *RET* | 3'UTR | C/T | T: 0.184 | T: 0.248 | C: 0.800 T: 0.200 | 500K genome-wide |
| 51 | rs3004214 | 42951704 | - | - | A/G | A: 0.102 | G: 0.486 | A: 0.444 G: 0.556 | 500K genome-wide |
| 52 | rs2505506 | 42965860 | - | - | A/G | A: 0.203 | G: 0.481 | A: 0.444 G: 0.556 | 500K genome-wide |
| 53 | rs7921281 | 42972658 | - | - | C/T | C: 0.083 | C: 0.145 | C: 0.156 T: 0.844 | 500K genome-wide |
| 54 | rs7893332 | 42972766 | - | - | G/T | G: 0.058 | G: 0.142 | G: 0.144 T: 0.856 | 500K genome-wide |
| 55 | rs7092548 | 42990811 | - | - | C/T | T: 0.059 | T: 0.152 | C: 0.856 T: 0.144 | 500K genome-wide |
| 56 | rs2435377 | 43003606 | - | - | A/G | A: 0.200 | G: 0.478 | A: 0.444 G: 0.556 | 500K genome-wide |
| 57 | rs12220534 | 43054312 | - | - | G/T | T: 0.191 | G: 1.000 | G: 0.567 T: 0.433 | 500K genome-wide |
| 58 | rs1879310 | 43054781 | - | - | A/G | A: 0.194 | G: 0.496 | A: 0.467 G: 0.533 | 500K genome-wide |
| 59 | rs7093409 | 43060073 | - | - | A/G | A: 0.190 | G: 0.499 | A: 0.433 G: 0.567 | 500K genome-wide |
| 60 | rs7090455 | 43073538 | - | - | C/T | C: 0.243 | C: 0.480 | C: 0.456 T: 0.544 | 500K genome-wide |
| 61 | rs2505526 | 43089897 | - | - | C/T | T: 0.257 | T: 0.472 | C: 0.544 T: 0.456 | 500K genome-wide |
| 62 | rs2503872 | 43113631 | - | - | A/G | A: 0.172 | A: 0.295 | A: 0.256 G: 0.744 | 500K genome-wide |
| 63 | rs2493650 | 43132991 | - | - | C/T | T: 0.144 | T: 0.157 | C: 0.889 T: 0.111 | 500K genome-wide |
| 64 | rs11238510 | 43133191 | - | - | C/T | T: 0.145 | T: 0.157 | C: 0.886 T: 0.114 | 500K genome-wide |
| 65 | rs2503875 | 43134055 | - | - | A/G | A: 0.472 | A: 0.456 | A: 0.556 G: 0.444 | 500K genome-wide |
| 66 | rs2493654 | 43140496 | - | - | A/C | A: 0.440 | A: 0.466 | A: 0.422 C: 0.578 | 500K genome-wide |
| 67 | rs2460535 | 43141386 | - | - | A/G | G: 0.276 | G: 0.308 | A: 0.667 G: 0.333 | 500K genome-wide |
| 68 | rs2243492 | 43169364 | - | - | C/T | T: 0.383 | T: 0.390 | C: 0.578 T: 0.422 | 500K genome-wide |
